# Viral Loads and Disease Severity in Children with Rhinovirus-Associated Illnesses

**DOI:** 10.3390/v13020295

**Published:** 2021-02-13

**Authors:** Maria I. Sanchez-Codez, Katherine Moyer, Isabel Benavente-Fernández, Amy L. Leber, Octavio Ramilo, Asuncion Mejias

**Affiliations:** 1 Division of Pediatric Infectious Diseases, Puerta del Mar University Hospital, Av. Ana de Viya, 21, 11009 Cádiz, Spain; mscodez1990@gmail.com; 2 Division of Pediatric Infectious Diseases, Inova Children’s Hospital, Falls Church, VA 22042, USA; kmoyer@psvcare.org; 3 Department of Paediatrics, Puerta del Mar University Hospital, 11009 Cádiz, Spain; isabenavente@gmail.com; 4 Biomedical Research and Innovation Institute of Cádiz (INiBICA) Research Unit, Puerta del Mar University Hospital, 11009 Cádiz, Spain; 5 Area of Paediatrics, Department of Child and Mother Health and Radiology, Medical School, University of Cádiz, 11003 Cádiz, Spain; 6 Department of Laboratory Medicine, Nationwide Children’s Hospital, Columbus, OH 43205, USA; Amy.Leber@nationwidechildrens.org; 7 Division of Infectious Diseases, Department of Pediatrics, Center for Vaccines and Immunity Nationwide Children’s Hospital—The Ohio State University College of Medicine, Columbus, OH 43205, USA; Octavio.Ramilo@nationwidechildrens.org; 8 Department of Pharmacology and Pediatrics, Malaga Medical School, Malaga University, 29010 Malaga, Spain

**Keywords:** rhinovirus, respiratory tract diseases, signs and symptoms, respiratory, infections, viral load, children

## Abstract

The role of rhinoviruses (RVs) in children with clinical syndromes not classically associated with RV infections is not well understood. We analyzed a cohort of children ≤21 years old who were PCR+ for RV at a large Pediatric Hospital from 2011 to 2013. Using univariate and multivariable logistic regression, we analyzed the associations between demographic, clinical characteristics, microbiology data, and clinical outcomes in children with compatible symptoms and incidental RV detection. Of the 2473 children (inpatients and outpatients) with an RV+ PCR, 2382 (96%) had compatible symptoms, and 91 (4%) did not. The overall median age was 14 months and 78% had underlying comorbidities. No differences in RV viral loads were found according to the presence of compatible symptoms, while in children with classic RV symptoms, RV viral loads were higher in single RV infections versus RV viral co-infections. Bacterial co-infections were more common in RV incidental detection (7.6%) than in children with compatible symptoms (1.9%, *p* < 0.001). The presence of compatible symptoms independently increased the odds ratio (OR, 95% CI) of hospitalization 4.8 (3.1–7.4), prolonged hospital stays 1.9 (1.1–3.1), need for oxygen 12 (5.8–25.0) and pediatric intensive care unit (PICU) admission 4.13 (2.0–8.2). Thus, despite comparable RV loads, disease severity was significantly worse in children with compatible symptoms.

## 1. Introduction

Human rhinoviruses (RVs) are the most common etiologic agents of acute respiratory tract infections in children and adults. RVs are commonly associated with the common cold, being responsible for over one-half of all upper respiratory tract infections (URTI) across all age ranges [1]. More recently, RVs have also been implicated in lower respiratory tract infections such as bronchitis, community-acquired pneumonia [1,2,3,4,5,6,7] bronchiolitis [1,4,8], and asthma exacerbations [1,4].

In the last two decades, the implementation of sensitive molecular tools for the diagnosis of RV infections has largely broadened the understanding of RV associated illnesses and has shown that RV can be detected in asymptomatic subjects [2,3,9,10,11]. In fact, rates of RV detection in asymptomatic children are higher than in adults ranging from 15% to 50% [11,12,13]. In addition, in the clinical setting, it is not uncommon to identify RV in combination with other respiratory viruses or in patients presenting with clinical syndromes that appear to be unrelated to classic RV infections, such as bone and joint infections or trauma. The high rates of RV detection in subjects presenting without compatible symptoms or who are completely asymptomatic have raised questions regarding the role of RV in certain clinical scenarios. Previous studies have attempted to define the significance of RV viral loads in asymptomatic subjects and to identify clinically relevant cut-off values for viral loads to help attribute RV causality in symptomatic patients [10,13,14]. Nevertheless, the role of RV detection in children with clinical syndromes not commonly associated with RV infections, and whether RV viral loads differ from those of children with classic RV-associated illnesses is not known.

The aim of the present study was to define demographic characteristics, clinical presentations, RV viral loads, viral and bacterial co-infections, and clinical outcomes in a convenience sample of children with RV-associated illnesses.

## 2. Materials and Methods

### 2.1. Study Design and Definitions

A retrospective cross-sectional study of a convenience sample of inpatients and outpatients who were ≤21 years old and tested positive for RV by PCR in respiratory samples obtained from 28 July 2011 to 31 December 2013 at Nationwide Children’s Hospital (NCH). NCH is the only tertiary care Children’s Hospital in Columbus (Ohio), and it is affiliated with The Ohio State University.

Patients were tested at the discretion of the attending physician, and PCR for seven respiratory viruses (RV, respiratory syncytial virus (RSV), parainfluenza virus (PIV), influenza virus (IV) A and B, adenovirus (ADV), and human metapneumovirus (hMPV)) ordered as a panel. Respiratory samples were processed at NCH Microbiology Laboratory following validated procedures. Respiratory samples tested included nasopharyngeal swab samples, bronchoalveolar lavage (BAL), and endotracheal tube specimens. The RV reverse transcription-PCR (RT-PCR) used targeted the 5´noncoding region (or 5´UTR) of RV as described [15]. Semiquantitative RV viral loads were reported as C*_T_* values. C*_T_* value > 40 were considered negative. Molecular typing of RV was not performed.

For children that were evaluated for the same episode in the outpatient and inpatient settings, only the hospitalization episode was analyzed. Similarly, for patients with multiple RV-associated hospitalizations, only the first admission during the same respiratory season was included. The seasonal pattern of RV infections was analyzed only in hospitalized patients due to the lack of consistent data in outpatients. We excluded patients > 21 years of age, those with no electronic health care records (EHR), or those who lacked complete clinical data.

Children in whom RV was identified were classified into two groups according to their clinical presentation—children with compatible symptoms (RV symptoms group) and those presenting with conditions in whom RV was not thought to play a role in the clinical presentation (RV incidental group). A compatible clinical presentation was defined by the presence of respiratory symptoms, fever, and/or gastrointestinal symptoms [1,16,17,18]. Children with RV detection without any of these clinical manifestations were included in the RV incidental group. Most of these children were tested as part of pre-screening procedures.

### 2.2. Data Collection

The following information was extracted from electronic health care records (HER), deposited into an electronic secured database (REDCap), and manually reviewed by two independent investigators. Briefly, we collected demographic and epidemiologic factors, including the presence of comorbidities and diagnosis. Microbiology tests performed included detection of other respiratory viruses in respiratory samples by PCR and bacterial cultures performed in blood, cerebrospinal fluid (CSF), urine, BAL, lower respiratory samples, and cystic fibrosis (CF) upper respiratory samples. Parameters of disease-severity were also analyzed and included the need for hospitalization, and in hospitalized patients, duration of hospitalization, need for and duration of supplemental oxygen (nasal cannula, non-invasive ventilation, or intubation), need for pediatric intensive care unit (PICU) admission, and duration of PICU stay. These are standard parameters of disease severity used in previous studies [19,20,21]. For analysis purposes, in children with more than one respiratory-related diagnosis at presentation or in those with multiple comorbidities, the most severe and relevant underlying disease was selected. The study was approved by the NCH Institutional Review Board.

### 2.3. Statistical Analysis

Qualitative variables were expressed as frequency and percentages. Quantitative variables were tested for normality and were reported as means and standard deviation (SD) if they followed a normal distribution, or as medians and 25–75% interquartile ranges (IQR) if data did not follow a normal distribution. For bivariate analysis, a chi-squared test or two-tailed Fisher’s exact tests were used to compare proportions, and t-test or Kruskal–Wallis tests for continuous data. We performed binary logistic regression to study the relationships between clinical factors (covariates) and outcome variables (need for hospitalization, PICU, length of stay, need for supplemental oxygen) and performed a post-estimation analysis of the univariate logistic regression models generated, with the estimation of the optimal cut- off values for those outcomes. Next, we performed multivariable logistic regression and included covariates that were significant in bivariate models or clinically relevant in relation to the clinical outcomes mentioned above. Two-sided *p*-values < 0.05 were considered statistically significant. Statistical analyses were performed using STATA 16.0. (StataCorp, College Station, TX, USA).

## 3. Results

### 3.1. Study Patients, Diagnoses, and Seasonality of RV Infections

**Figure 1:**
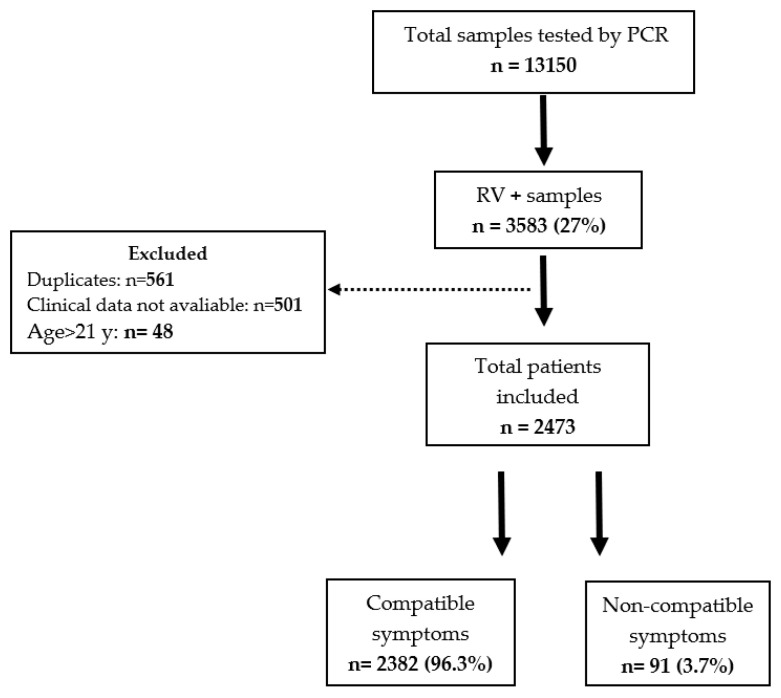
Flow diagram depicting the selection of study patients.

From 28 July 2011 to 31 December 2013, 13,150 respiratory samples from children evaluated in the inpatient and outpatient setting were analyzed by PCR at NCH Microbiology Laboratory (Figure 1). Of those, 3583/13150 (27%) children tested positive for RV. We excluded duplicate samples collected during the same episode, patients with no clinical data available, and children older than 21 years old, resulting in 2473 unique patients with confirmed RV detection. Of those, 2382/2473 (96.3%) children had compatible symptoms (RV symptoms group), and 91/2473 (3.7%) did not have symptoms compatible with RV infection (RV incidental group). Within the cohort, 2099 children were hospitalized (85%), and 374 (15%) were managed as outpatients.

The majority of children included in the RV symptoms group presented with a respiratory-related diagnosis (2295/2382 (96.3%); Table 1). The most common respiratory diagnoses were upper respiratory tract infections (URTI), followed by bronchiolitis and asthma exacerbations. The proportion of children with a lower respiratory tract infection (LRTI)-related diagnosis (bronchiolitis, asthma, pneumonia; *n* = 1274) was higher than those with URTI (*n* = 680; *p* < 0.001), with no differences in RV viral loads between groups 25.02 (21.90–28.94) for URTI versus 25.28 (22.13–29.26) for LRTI-related diagnoses; *p* = 0.245. Gastrointestinal (GI) symptoms (diarrhea, vomiting, dehydration, or decreased oral intake) were present in 530/2382 (22.25%) patients, the majority in combination with respiratory symptoms (460 with respiratory and GI symptoms; 70 (2.9%) with GI symptoms exclusively). Fever with no other symptoms was the clinical presentation in 17/2382 (0.7%) of children.

Children included in the RV incidental group had a variety of clinical presentations (Table 2), including cardiovascular manifestations, gastroesophageal reflux, Kawasaki Disease, trauma, etc.

**Figure 2:**
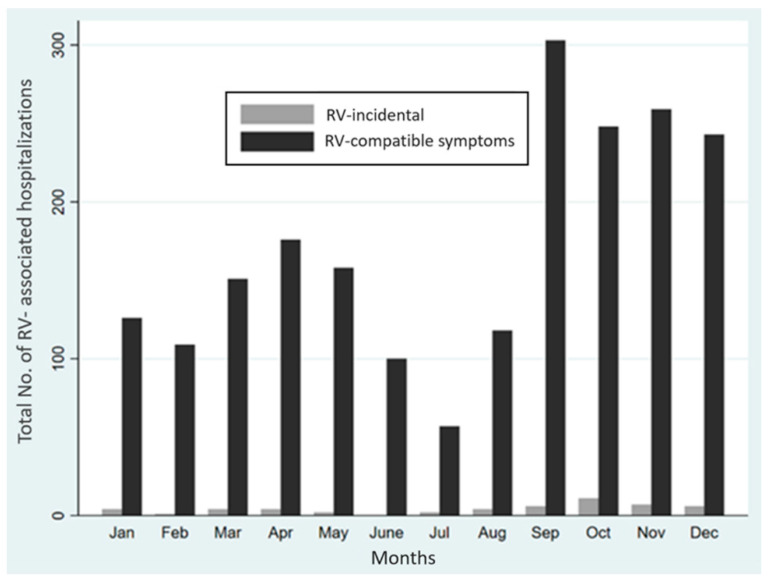
Seasonality of RV-associated hospitalizations.

The seasonality of RV in hospitalized children over the 30-month (2011–2013) study period is depicted in Figure 2. Of the 2099 hospitalized children with RV detection, 2048 had compatible symptoms and 51 were included in the RV incidental group. The number of RV cases identified occurred steadily throughout the year, with over 100 patients per month except for June and July. RV detection peaked in the last quarter of the year. Overall, most of the cases were diagnosed in September 309/2099 (14.72%) without differences between patients with compatible symptoms and those with RV incidental detection 303/2048 (14.79%) versus 6/51 (11.76%); *p* = 0.69. Similarly, there were no variations in seasonal patterns in the rest of the year according to the clinical presentation.

The horizontal axis shows the months of the year for RV detection in patients with compatible (black) symptoms and RV incidental detection. The vertical axis shows the total number of RV-associated hospitalizations in both groups.

### 3.2. Demographic Characteristics and Underlying Diseases

Demographic characteristics and underlying diseases in children with RV-associated illnesses are summarized in Table 3. Overall, the median age was 14.3 (3.9–49) months with no differences in age, sex, or race between children with RV-associated symptoms and those with RV incidental detection. More than two-thirds of children in both groups had underlying chronic conditions. Asthma/atopy/allergy were the most common underlying conditions in children with compatible symptoms (42.15%) versus those with incidental RV detection (18.31%; *p* = 0.012). On the other hand, congenital heart disease was significantly more common in children with incidental RV detection than in those with compatible symptoms (14.08% versus 4.40% respectively; *p* = 0.007). All children with CF had compatible symptoms.

### 3.3. Viral and Bacterial Co-Infections

Rates and types of RV viral co-infections and results from bacterial cultures are summarized in Table 4. Respiratory viral co-infections were more frequent in patients with compatible symptoms than in children with incidental RV detection (22.3% versus 10.9%; *p* = 0.010) with adenovirus being the most common viral pathogen co-detected in both groups (9% and 10%, respectively). RSV and influenza A and B were co-detected in 7.7% and 0.5% of children, respectively, and exclusively in children with compatible symptoms.

**Figure 3:**
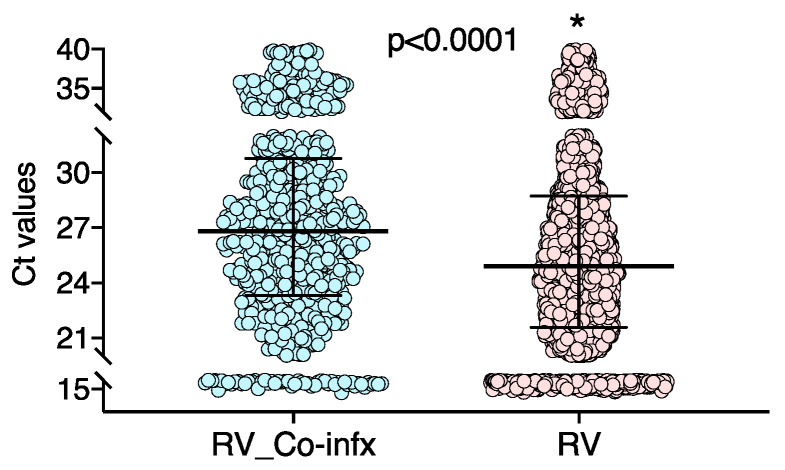
Comparison of RV viral loads in children compatible symptoms and RV mono-infections versus RV viral co-infections. RV_Co-infx: RV co-infections with another respiratory virus.

We also analyzed whether RV viral loads differed according to the clinical presentation and co-detection of other respiratory viruses. We did not find significant differences in RV loads in children with compatible symptoms versus those with incidental RV detection (Table 4). In children with compatible symptoms, RV viral loads were significantly higher (lower Ct values) than in children with RV mono-infections (*n* = 1850; 24.81 (21.53–25.88) C*_T_* values) than in those with RV viral co-infections (*n* = 532; 26.85 (23.41–30.68) C*_T_* values; *p* = 0.0001; Figure 3). These differences were not observed in children with incidental RV detection in whom RV viral loads in RV mono-infections and co-infections were not significantly different, i.e., (25.47 (22–29.07)) and (26.51 (23.9–27.3), respectively; *p* = 0.442).

Regarding bacterial cultures, children with compatible symptoms had significantly lower rates of bacteremia (1.95% versus 7.69%, respectively; *p* = 0.015) and bacterial meningitis (0.85% versus 7.7%, respectively; *p* = 0.028) than those in the RV incidental group. RV viral loads in children with compatible symptoms and bacterial co-infections were comparable to those without bacterial infections 24.9 (21.2–29.1) and 25.42 (22.04–29.3) C*_T_* values respectively; *p* = 0.129). Similarly, children with RV incidental detection had comparable RV viral loads irrespective of bacterial co-infections 26.21 (24.32–29.27) and 26.12 (21.66–30.25) for children with and without bacterial co-infections respectively; *p* = 0.41).

### 3.4. Disease Severity Parameters in Children with Compatible Symptoms and Incidental RV Detection

Disease severity in children within the RV symptoms and RV incidental detection groups is summarized in Table 5. Overall, disease severity parameters were worst in children with RV-associated symptoms than in those with RV incidental detection. The proportion of hospitalized children in the RV symptoms group was higher than in the RV incidental cohort (85.98% versus 56.4%; *p* = 0.0001; OR, 95% CI: 4.8 (3.1–7.4)). In addition, children with compatible symptoms had more prolonged duration of hospitalizations, higher rates of PICU admission, and more frequent need for supplemental oxygen than children with incidental RV detection. Duration of supplemental oxygen via non-invasive or invasive mechanical ventilation was comparable between groups.

Lastly, we performed multivariable logistic regression analyses to determine whether symptomatic or incidental RV detection was associated with the outcomes of care identified as significantly different in bivariate analyses, namely, need for hospitalization, need for supplemental oxygen, PICU admission, and length of stay, which was dichotomized by the median in ≤1.8 days and >1.8 days. We included as covariates in the models’ demographic factors, RV viral loads, viral co-infections, and presence of comorbidities (Table 6).

Age and presence of RV-associated symptoms were consistently and independently associated with all outcomes of care. In addition, higher viral loads (lower Ct values) increased the risk of PICU admission while viral co-infections were associated with decreased odds of hospitalization. Underlying diseases increased the odds of longer length of stay adjusted for all other covariates.

## 4. Discussion

In the present study, we analyzed the differences in demographic factors, microbiology, and clinical outcomes in children with classic RV-associated symptoms and those with incidental RV detection. We found that irrespective of their clinical presentation, a high proportion of children had underlying chronic medical conditions, with asthma being more prevalent in children with RV-compatible symptoms. While we did not find any differences in RV viral loads according to their clinical presentation, RV loads in children with compatible symptoms and single RV detection were significantly higher than in those with RV viral co-infections. On the other hand, severe bacterial infections (SBI) defined as bacteremia and meningitis were more frequent in children with RV incidental detection than with classic RV-associated illnesses. Overall, this study illustrates the relevant role of RV associated illnesses beyond asthma and the importance of combining demographic and clinical information to facilitate the interpretation of RV detection in the clinical setting.

To define the role of RV in clinical disease severity, different studies have either compared clinical outcomes in children with RV infections versus infections with other respiratory viruses [22], or whether differences in severity exist according to RV genotypes [3,7,16,17,23,24,25,26,27,28]. In addition, the high prevalence of RV detection has stimulated investigators to address the role of RV in asymptomatic subjects [10,13,29] and also in patients hospitalized with respiratory symptoms [27]. In the present study, we considered a broader approach and focused our efforts on understanding the significance of RV detection in a large cohort of children with a wide variety of clinical presentations. We analyzed whether differences existed in demographic parameters, virology characteristics, and clinical disease severity in both outpatients and inpatients with and without classic RV-associated illnesses.

The classic clinical presentations associated with RV infections include URTI, bronchiolitis, and asthma exacerbations being the most common, followed by tracheitis, laryngitis, otitis media, or fever [1,3,4,14,30,31]. In the present study, in addition to asthma, which was the most common diagnosis, we identified a large proportion of children with involvement of the lower respiratory tract, with diagnoses of bronchiolitis, pneumonia, and LRTI. Together as a group, these children represented 37% of all those in the RV compatible illness group. Studies have also identified RV in stool samples from patients with gastrointestinal syndromes [32], suggesting it may play a role in their clinical presentation [27]. In addition, decreased oral intake was identified as a relevant GI symptom of RV associated illnesses at all ages [16,17,18,19]. For these reasons and although the majority of children with GI manifestations in our cohort also had respiratory symptoms, children with GI symptoms were included in the group of children with compatible RV symptoms.

In agreement with previous studies, the prevalence of underlying chronic conditions in children with RV detection was high and independently associated with worse clinical outcomes, suggesting their higher vulnerability to severe RV infections [2,16,19,28]. Of those pre-existing conditions, and as reported by several previous studies, asthma was the most prevalent underlying disease identified both in children with classic RV-associated illnesses and in those with incidental RV detection [8,13,25,27,33,34]. In agreement with previous studies, we identified RV in children with CF exacerbations [4,8,31,35]. In fact, RV was associated with an earlier decline in lung function in patients with CF [8]. We also found that non-pulmonary diseases were commonly present in children with incidental RV detection, such as congenital heart diseases, which may indicate the inability of these patients to properly clear RV from the respiratory tract [2]. However, it may also reflect a clinical decision for enhancement of testing in this high-risk population. Prematurity has also been identified as a risk factor for severe RV respiratory infections, including community-acquired pneumonia, and was present in 13% of children in our study [6,18,36]. Immunocompromised patients are at a greater risk of severe RV infections [8,37]. On the other hand, incidental detection of RV in these patients has been reported. In agreement with previous studies, rates of RV detection in immunocompromised children without the classic RV-associated symptoms was not uncommon [38].

Rates of RV co-infections with other respiratory viruses were more common in children with compatible symptoms. In line with prior studies, RSV was one of the most frequent virus co-detected in patients with RV-associated respiratory illnesses [2,7,19,25], but not in those with incidental RV detection [24,33]. On the other hand, adenovirus was the most common co-pathogen identified in both children with compatible symptoms and in children with incidental RV detection [16,26]. The number of children with influenza co-detections was low and similar to other reports [16,25], suggesting the possibility of an interference mechanism [16,19].

Most of the studies that have evaluated the role of RV viral loads in RV-associated illnesses found higher viral loads in symptomatic than in asymptomatic patients [10,11,12,13,14]. In our study, children included in the incidental detection group, rather than asymptomatic, were hospitalized with diseases not presenting with the classic RV-associated symptoms, which may explain the differences observed between our study and previous reports. Investigators have also attempted to define the role of RV viral loads and/or identify viral load cut-offs predictive of clinical disease severity with different results [10,12,13,14,27,39,40]. While higher RV loads were associated with higher clinical disease severity scores, increased risk of LRTI, and respiratory failure in children [27,40,41], other studies have not confirmed these associations [39,42], which may be explained by differences in study design and patients populations included.

Overall, bacterial co-infections in children with RV infections are low, although rates may vary according to the clinical presentation [7,16,43,44]. Higher rates of SBI were reported in patients with LRTI requiring PICU care [45] and in the young infant with fever without a source, in whom RV detection is not associated with a decreased risk of SBI, such as urinary tract infections and bacteremia [46]. In our study, the median age of study subjects was 14 months, and rates of bacterial co-infections were higher in patients with incidental RV detection, further suggesting that the bacterial pathogens identified in patients with SBI were responsible for the clinical presentation and RV detection a true incidental finding.

Different clinical outcomes, such as the need for supplemental oxygen, PICU care, length of hospital stay, or need for mechanical ventilation have been used to assess the severity of RV-associated illnesses in children [5,16,18,22,23,24,25,26]. In our study, we found that children presenting with RV illnesses and compatible symptoms had consistently greater disease severity, as assessed by the outcomes of care mentioned above and adjusted for all other covariates, highlighting the increased risk of enhanced disease in these children.

Our study has limitations. Its retrospective design limited our ability to collect precise information related to factors, such as a family history of asthma or second-hand smoke exposure that may have influenced the clinical outcomes assessed. In addition, host immune responses were not assessed and thus the contribution of RV to the illnesses identified could not be fully elucidated. Duration of illness at the time of sample collection might have influenced RV viral loads, but this parameter was not collected. Despite this limitation, we found that children with compatible symptoms and RV mono-infections had higher RV viral loads than those with RV viral co-infections. Lastly, the study was performed at a single center, and over a limited duration, and the convenience sample of patients tested might represent the subset of patients with more severe disease. Nevertheless, NCH is the only pediatric hospital in central Ohio and serves a large and varied population of pediatric patients, which allowed us to conduct multivariable analyses adjusted for these confounders.

In conclusion, this study represents one of the largest case series to date describing the spectrum of RV associated illnesses in children, further comparing children with common symptoms with those with RV incidental detection. While incidental detection of RV in patients with non-compatible symptoms was low, children with classic RV illnesses had worse disease severity assessed by different outcomes of care analyzed. RV viral loads were comparable in children with classic RV symptoms and those with RV incidental detection. Whether RV played a role in the clinical presentation of these children irrespective of their clinical classification deserves further studies linking RV detection and viral loads with host immune responses. In summary, we found that a detailed classification of pediatric patients with RV detection based on clinical presentations, viral loads, and rates of viral and bacterial co-infections shed light on the different RV clinical phenotypes and may help with patient classification in the clinical setting.

## Figures and Tables

**Table 1:** Respiratory diagnosis in patients with compatible symptoms (*n* = 2295).

Diagnosis	*N* (%)
URTI	680 (29.62)
Bronchiolitis	483 (21.04)
Asthma exacerbation	454 (19.80)
Pneumonia	228 (9.93)
LRTI	109 (4.74)
Respiratory failure	160 (7.00)
Apnea	5 (0.20)
Hypoxemia	11 (0.47)
CF exacerbation	46 (2.00)
Ventilator-associated pneumonia	47 (2.04)
AOM/sinusitis	26 (1.13)
Airway malacia exacerbation	1 (0.04)
Other	2 (0.09)

Data presented as absolute numbers with percentages in parenthesis. Other included two children with an exacerbation of chronic pulmonary heart disease. URTI, upper respiratory tract infection; LRTI, lower respiratory tract infection; CF, cystic fibrosis; AOM, acute otitis media.

**Table 2:** Diagnosis in patients with incidental rhinovirus (RV) detection (*n* = 91).

Diagnosis	*N* (%)
Cardiovascular	24 (26.37)
GERD	19 (20.87)
Neurological disorders	13 (14.28)
Immunocompromised	7 (7.70)
Kawasaki Disease	6 (6.59)
Seizures	5 (5.49)
Routine health examination	4 (4.40)
Hematologic disease	4 (4.40)
Syndromes	2 (2.20)
Sepsis/shock	2 (2.20)
Neuromuscular disorders	1 (1.10)
Endocrine disorders	1 (1.10)
Prematurity	1 (1.10)
Surgery	1 (1.10)
Trauma	1 (1.10)

Data presented as frequency (percentage). GERD, gastroesophageal reflux disease.

**Table 3:** Demographic characteristics and underlying diseases of children with compatible and non-compatible symptoms.

	RV Symptoms Group (*n* = 2382)	RV Incidental Group (*n* = 91)	*p* Value
**Demographic Characteristics**			
Age, (months)	14.35 (3.9–49.3)	14.10 (3.8–38.1)	0.844
Sex, n (%) male	1404 (58.94)	55 (60.44)	0.776
Race or ethnic group, n (%)			0.449
White	1320 (55.42)	58 (63.74)	
Black or African American	641 (26.91)	19 (20.88)	
Biracial	88 (7.89)	5 (5.49)	
Other	233 (9.78)	9 (9.89)	
Underlying diseases, n (%)	1886 (79.17)	71 (78.02)	0.031
Asthma/atopy/allergy	795 (42.15)	13 (18.31)	0.012
Genetic syndromes	256 (13.57)	18 (25.35)	0.096
Prematurity	251 (13.31)	9 (12.68)	0.774
Gastrointestinal diseases	82 (4.34)	4 (5.63)	0.734
Congenital heart disease	83 (4.40)	10 (14.08)	0.007
Neurological disorders	58 (3.08)	3 (4.23)	0.695
Cystic fibrosis	46 (2.44)	0 (0.00)	-
Respiratory tract morbidity ^a^	55 (2.92)	2 (2.82)	0.891
Immunodeficiencies	35 (1.86)	4 (5.63)	0.070
Hematologic disease	23 (1.22)	1 (1.41)	0.943
Other ^b^	202 (10.71)	7 (9.86)	0.735

Continuous variables are expressed as medians 25–75% interquartile ranges (IQR) and categorical data as frequency (%). Numbers in bold indicate significant *p* values. ^a^ Respiratory tract morbidity included neonatal intubation for 10 or more days, in relation to meconium aspiration syndrome, gastroschisis, or congenital diaphragmatic hernia. Other diseases included were tracheoesophageal fistula, pulmonary hypertension, spontaneous pneumothorax, airway malacia, subglottic stenosis, cleft palate, and Pierre–Robin sequence. ^b^ Endocrine, ophthalmologic, osteoarticular, and genitourinary underlying diseases.

**Table 4:** Microbiology data of children with compatible symptoms versus incidental detection of RV.

	RV Symptoms Group (*n* = 2382)	RV Incidental Group (*n* = 91)	*p* Value
Viral co-infections ^a^	532/2382 (22.33)	10/91(10.99)	0.010
Adenovirus	241 (10.12)	8 (8.79)	**0.679**
RSV	185 (7.77)	0 (0)	**0.006**
Parainfluenza	98 (4.12)	1 (1.10)	**0.150**
hMPV	53 (2.23)	2 (2.20)	**0.986**
Influenza A	9 (0.38)	0 (0)	**0.557**
Influenza B	6 (0.25)	0 (0)	**0.632**
RV viral loads	25.2 (22.05–29.04)	25.6 (22.09–29.07)	**0.934**
Bacterial co-infections ^b^			
Blood culture	21/1076 (1.95)	3/39 (7.69)	**0.015**
Cerebrospinal fluid culture	2/235 (0.85)	1/13 (7.69)	**0.028**
Urine culture	41/566(7.24)	1/17 (5.88)	**0.831**
AL culture	14/38 (34.84)	3/3 (100)	**0.064**
Lower respiratory culture	91/243 (37.44)	3/4 (75)	**0.156**
Cystic fibrosis culture	11/32 (34.38)	0/0 (0)	**-**
Other cultures ^c^	45/212 (21.23)	2/12 (16.67)	**0.706**

Numbers in bold indicate significant *p* values. ^a^ Co-infections indicate the presence of more than one respiratory virus: rhinovirus (RV), respiratory syncytial virus (RSV), parainfluenza virus, influenza virus A and B, adenovirus, or human metapneumovirus (hMPV). ^b^ *n* positive cultures/*N* Total (%). Data represent the number of positive cultures/total number of cultures performed. Numbers in parentheses specify the percentage of positive cultures. ^c^ Other cultures included bacterial and fungal cultures performed from sterile (pleural fluid, body fluid, and catheter) and non-sterile sites (genital, eye, stool, wound, throat/nasopharyngeal). BAL, bronchoalveolar lavage.

**Table 5:** Univariate associations of disease severity parameters according to clinical presentation.

	RV Symptoms Group (*n* = 2382)	RV Incidental Group (*n* = 91)	Odds Ratio (Upper-Lower CI)	*p* Value
Hospitalization	2048 (85.98)	51 (56.4)	4.80 (3.12–7.39)	0.0001
PICU	743 (31.19)	9 (9.89)	4.13 (2.06–8.26)	**0.0001**
Length of stay ^a^	2.4 (1.5–4.7)	1.7 (1–3.1)	1.90 (1.14–3.17)	**0.013**
Length of stay in PICU ^b^	2.4 (1.4–4.7)	1.6 (1.2–2.6)	3.30 (0.82–13.32)	**0.093**
Suplemental oxygen				
Requirement, *n* (%)	1278 (53.65)	8 (8.79)	12.01(5.78–24.92)	**0.0001**
Duration (days) ^c^	2 (1–4)	2.5(1.5–6)	0.95 (0.19–4.74)	**0.953**
Oxygen administration via				
Nasal Cannula	736 (57.59)	5 (62.50)	1.71 (0.40–7.24)	**0.461**
NIV	285 (22.30)	0 (0)	-	**-**
Mechanical ventilation	257 (20.11)	3 (37.50)	0.58 (0.13–2.45)	**0.461**

Data presented as frequency (percentage). Numbers in bold indicate significant *p* values. ^a^ Prolonged hospital stay if greater than the median length of stay, which was 1.8 days. ^b^ Prolonged pediatric intensive care unit (PICU) stay if greater than the median length of PICU stay, which was 1.9 days. ^c^ Prolonged duration of supplemental oxygen if greater than median O2 supplementation that was 4 days. NIV, non-invasive ventilatory support; PICU, pediatric intensive care unit.

**Table 6:** Multivariable logistic regression of risk factors associated with disease severity.

	Hospitalization (OR, 95% CI) *p*-Value		PICU (OR, 95% CI) *p*-Value		Length of Stay (OR, 95% CI) *p*-Value		Supplemental Oxygen (OR, 95% CI) *p*-Value	
Age	1.06 (1.03–1.09)	**0.0001**	1.07 (1.05–1.09)	**0.0001**	1.10 (1.08–1.13)	**0.0001**	1.08 (1.06–1.10)	**0.0001**
Female	0.90 (0.72–1.13)	0.386	0.96 (0.80–1.14)	0.662	0.98 (0.82–1.17)	0.838	0.94 (0.80–1.11)	0.494
RV viral loads	0.99 (0.97–1.01)	0.319	0.97 (0.95–0.99)	**0.004**	0.98 (0.96–1.00)	0.090	0.99 (0.97–1.00)	0.087
RV viral co-infections ^a^	0.73 (0.57–0.95)	**0.021**	1.2 (0.96–1.4)	0.094	1.04 (0.84–1.29)	0.682	1.11 (0.91–1.35)	0.314
Compatible symptoms	4.99 (3.23–7.73)	**0.0001**	3.9 (1.95–7.92)	**0.0001**	2.03 (1.20–3.42)	**0.008**	11.86 (5.68–24.76)	**0.0001**
Underlying diseases	1.01 (0.98–1.04)	0.650	0.98 (0.96–1.00)	0.121	1.02 (1.00–1.04)	**0.030**	0.98 (0.96–1.00)	0.059

Odds ratios (ORs) and 95% confidence interval (CI). Numbers in bold indicate significant *p* values. ^a^ Viral co-infection indicates the detection of more than one respiratory virus apart from RV: respiratory syncytial virus (RSV), parainfluenza virus, influenza virus A and B, adenovirus, or hMPV.

## Data Availability

Deidentified per patient clinical data can be provided upon request.
